# The Spatial–Temporal Effects of Bacterial Growth Substrates on Antibiotic Resistance Gene Spread in the Biofilm

**DOI:** 10.3390/antibiotics12071154

**Published:** 2023-07-06

**Authors:** Shuzhen Liu, Bingwen Liu, Yin Zhu, Yong Qiu, Bing Li

**Affiliations:** 1 School of Energy and Environmental Engineering, University of Science and Technology Beijing, Beijing 100083, China; lsz1671@163.com (S.L.); liubingwen529529@163.com (B.L.); 2 School of Environment, Tsinghua University, Beijing 100084, China; zyfreely@126.com

**Keywords:** antibiotic resistance genes, biofilm formation, agarose microfluidic chip, bacterial growth substrates, plasmid conjugation

## Abstract

Biofilm is considered as the hotspot of antibiotic resistance gene (ARG) dissemination. Bacterial growth substrates are important factors for biofilm formation, but its spatial–temporal effects on ARG spread in biofilm is still unclear. In this study, microfluidics combined with microscopic observation were used to reveal spatial–temporal effects of bacterial growth substrates on ARG transfer at real time. The initial horizontal gene transfer events were found to be independent of substrate levels. However, subsequent transfer processes varied greatly depending on the availability of growth substrates. The proportion of transconjugants was much higher (~12%) when observed in substrate-rich regions (under the channel) at 24 h, followed by an exponential decline, with the distance far from the channel. Furthermore, three-dimensional observation revealed that vertical gene transfer influenced by the concentrations of bacterial growth substrates was important for ARG spread in biofilm. The transfer frequency was 8.2 times higher in the high substrate concentration (50×) compared to low concentration (0.5×) in simulated sewage, underscoring the substantial impact of bacterial growth substrate variability on ARG dissemination. This study is helpful for in-depth understanding of ARG dissemination through biofilms and indicates that reducing pollutant emission is important for ARG control in the environment.

## 1. Introduction

Bacteria adhere to various substrate surfaces resulting in complex biostructures enclosed in extracellular polymeric substances (EPS), which are called biofilms [1,2]. It is commonly accepted that biofilms occur in almost any submerged surfaces in both natural and artificial environments, as a suitable habitat for bacterial growth, metabolism and communication [3], due to the high-diversity and well-organized community, the composition of various chemical compounds and high mutagenicity. Biofilms in water environments are thought to be hotspots for horizontal gene transfer (HGT) of antibiotic resistance genes (ARGs) [4]. The main types of HGT include conjugation, transformation and transduction, which are mediated by mobile genetic elements (MGEs) such as plasmids. ARGs also can be transferred by vertical gene transfer (VGT). Many studies have shown that bacteria frequently exchange ARGs in biofilms [5,6]. The transfer rates within the biofilm community have been reported to be 700 times higher than planktonic bacteria [7]. Therefore, ARG dissemination through biofilms should be paid more attention to in the environment.

The formation of biofilm is a dynamic process, including a colonization stage of reversible adhesion, agglomeration stage of irreversible adhesion, mature stage, aging and re-plantation stage. During the process, the composition and diversity of biofilms are affected by a broad range of environment conditions (e.g., temperature [8], pH [9], dissolved organic carbon [10], bacterial growth substrates (including carbon source, nutrients, etc.) [11,12], shear stress [13] and salinity [14]) and adhered surfaces [15]. Among these environmental factors, bacterial growth substrates are most important for biofilm formation. Previous studies have confirmed that the growth near the biofilm–nutrient source interface is more active in mature biofilms generally [16,17]. Furthermore, bacterial growth substrates including nutrients have been found to be positively related to the abundance of ARGs in different environments, including wastewater [18], soil [19] and seawater [20], and could regulate the health risks of ARGs [21]. Therefore, bacterial growth substrates could play an important role in ARG spread in biofilm. As HGT and VGT happen during the biofilm formation, it is necessary to reveal how they influence ARG spread by spatial–temporal analysis.

Visualization is important in the detection and monitoring of biofilm formation; microfluidics, as an emerging technique for biofilm study, attract more and more attention [22]. Firstly, it allows the precise control of test conditions on the order of microliters [23]. Next, it allows the perfusion culture through different designed channels instead of excess substrates in the traditional system for days. In the process, fresh media are supplied continuously, and metabolic wastes are removed with the flow [24], resulting in chemical gradients. Additionally, it allows the in-situ control of cells in micrometers and real-time observation. Moreover, our previous studies have proved that microfluidics can provide a better tool for ARG transfer study, which could track the transfer processes by microscopes [25]. However, the previous studies used two-dimensional observation; it is hard to describe the detailed transfer dynamics in the biofilm. Therefore, combining microfluidics and three-dimensional (3D) observation should be developed for ARG transfer study in the biofilm.

In this study, a microfluidic chip with continuous flow was used to form different thicknesses of biofilm under the gradient of bacterial growth substrates; HGT firstly happened at a similar time, and the following ARG processes were obviously influenced by the substrate supply. The 3D observation revealed the important role of VGT for ARG spread. Furthermore, the transfer frequencies in the different substrate concentrations of simulated wastewater were also tested to compare with the observed results.

## 2. Results and Discussion

### 2.1. The Biofilm Formation with Different Thicknesses on the Microfluidic Chip

**Figure 1:**
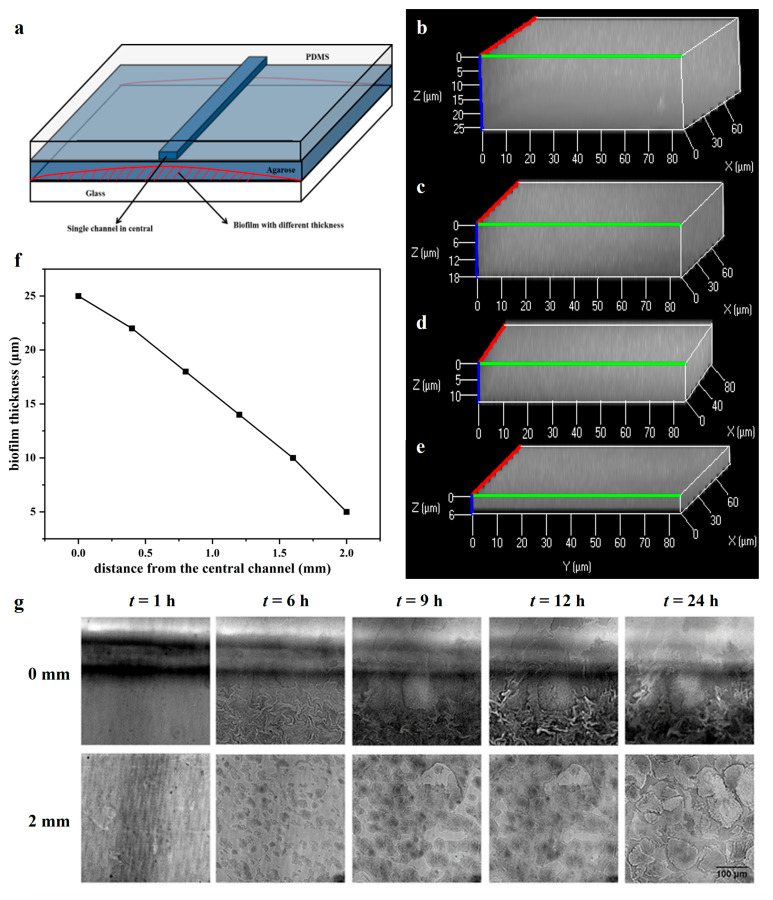
Biofilms with varying thicknesses in the microfluidic chip (**a**) supplied by LB medium. The distance from the central passage is (**b**) 0 mm; (**c**) 0.8 mm; (**d**) 1.6 mm; (**e**) 2 mm; (**f**) The change in biofilm thickness with the increase in distance from the central channel. (**g**) The process of biofilm formation under the central channel (0 mm) and away from the channel (2 mm). All pictures are adjusted according to the displayed scale bar in the bottom-right picture (100 µm).

The distribution of bacterial growth substrate (LB medium was used on the microfluidic chips) was heterogeneous across the agarose membrane; the thickness of the biofilm varied at different positions between the agarose and glass. Bacteria under the channel grew fast and formed thicker biofilm. Figure 1 shows the 3D images of biofilms at different positions taken by CLSM (100× objective). The thickest biofilm was as high as 25 µm, which formed under the central channel (Figure 1b), while the thinnest biofilm was only 5 µm, which was almost the single layer biofilm (Figure 1e). The thickness of biofilm became thinner with the increasing distance from the central channel (Figure 1f), i.e., with the decrease in substrate concentration, the growth of bacteria became weak. Therefore, compared with other methods such as using carriers [26], the microfluidic chip provided a simple way to form biofilms with varied thicknesses at one time for study in virtue of substrate diffusion in agarose membranes.

The process of biofilm formation on the microfluidic chip was tracked at different positions by the inverted microscope and is shown in Figure 1g. At the position of 0 mm from the central channel, the substrates were abundant, and bacteria grew very quickly, a monolayer biofilm formed at 6 h, and bacteria stacked to multilayers at 9 h and became thicker and thicker; while at the position of 2 mm, bacteria started to form small colonies at 6 h, single colonies grew larger and connected gradually, and kept in a monolayer but had not formed the thick biofilm covering the glass at 24 h. The results are in line with previous studies that the bacterial activity and growth rate in biofilm development are related to substrate availability [12,27]. There are studies shown that the biofilm formation is easily affected by substrate condition, including organic and inorganic matters [28,29]. In these studies, cell population always was observed to migrate, driven by chemical gradient (e.g., nutrient) [30], and bacteria tended to improve respiration and growth rates in substrate-rich areas [31].

### 2.2. The Transfer Processes of Antibiotic Resistance Plasmid under Different Substrate Conditions on the Chip

Conjugation, which is the plasmid DNA transfer between the bacterial cells by bacterial pilus contact, plays a dominant role in HGT. As conjugation between biofilm bacteria will depend intimately on the flow regime in which they are submerged [32], the microfluidic chip provided a suitable environment for conjugation. Because the agarose membrane was fixed between the channel and glass, and the flow rate set was stable and faint, biofilm formation would not be influenced by the flow; otherwise, the conjugation transfer rate can be affected by flow velocity indirectly, through the effect on bacterial density and biofilm formation [33]. As mentioned above, LB medium was injected from the central channel of the chip, resulting in different thicknesses of biofilm at different positions.

**Figure 2:**
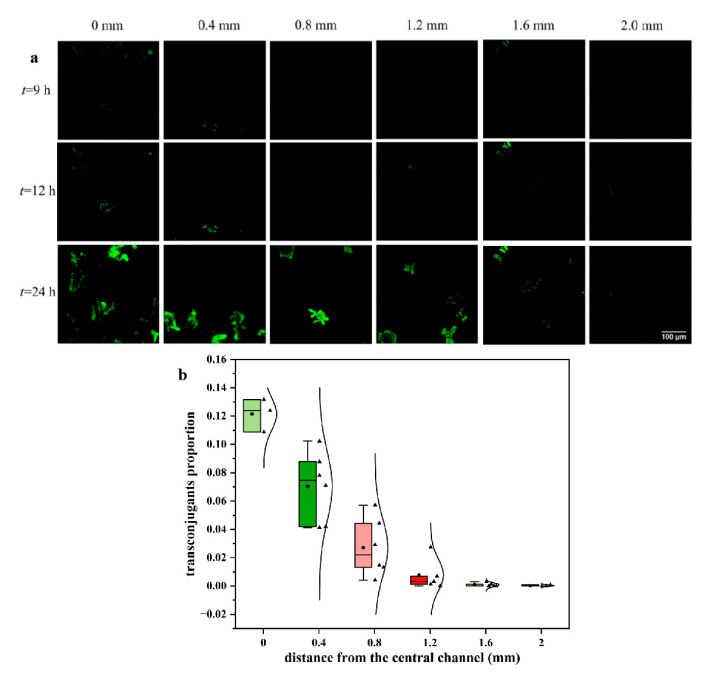
The transfer process of ARG at different positions on the chip supplied by LB medium: (**a**) Time-lapse images of ARG transfer (transconjugants occurred at 9 h). The scale bar represents 100 µm; (**b**) The proportion of transconjugants at different positions. In the boxplot, the horizontal line (from top to bottom) represents the maximum, upper quartile, median, lower quartile, and minimum values. The circles represent the average values. The triangles represent data points of parallel samples, and the curves represent the dispersion of the data.

During the process of biofilm formation, conjugation was also recorded at real time. Figure 2a showed the existence of transconjugants at each study position observed by the inverted fluorescence microscope. The transconjugants presented as green fluorescence because the plasmid pKJK5 transferred to the recipient bacteria and GFP expressed due to the lack of an inhibitor. The HGT events almost appeared at 9 h at different positions, which showed less influenced by the substrates. However, the following transfer processes varied greatly with different substrate supply. At the position 0 mm, the transconjugant area increased gradually, which was much larger compared with other positions at 24 h. Furthermore, the transconjugant proportions (*A*_PG_) were calculated as Equation (1) at different positions at 24 h, which were inversely correlated to the distance from the central channel (Figure 2b). The transconjugant proportion at the position 0 mm was about ~12%, while at the positions farther from the central channel (1.6 and 2.0 mm), very few transconjugants appeared (~0%). The results indicated VGT may be dominant after HGT occurred in the biofilm. The positive correlation between the biofilm thickness and transconjugant proportion revealed the possible driving forces of biofilm structure and substrate condition to promote ARG dissemination. Due to the complex structure, the inner transfer process in biofilms was also affected by the substrate supply. Our previous study proved that almost the same distribution of transconjugants occurred across all layers of the biofilm [34], so the transconjugant proportions could stand for how many transconjugants appeared in the whole biofilm. However, the detailed transfer process through the biofilm formation was not observed through two-dimensional (2D) observation.

### 2.3. The Contribution of VGT to the ARG Spread Revealed by the 3D Distribution Analysis in the Biofilm

**Figure 3:**
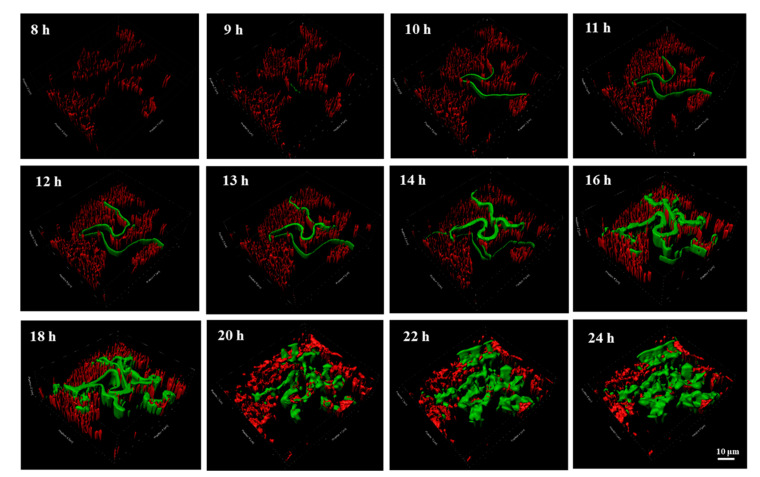
The 3D images recording the transfer process of ARG in the biofilms on the chip (studied position is under the central channel supplied by LB medium).

In order to reveal the transfer process across the different layers of the biofilm, the 3D distribution of transconjugants under the central channel was observed by a live cell imaging system, as shown in Figure 3. The transconjugants occurred at 9 h near the bacterial cluster and connected into a green band. With the bacterial growth, the band extended breadthwise and lengthwise. In the horizontal direction, the distribution of transconjugants had a certain randomness, which was mainly attributed to HGT. While in the vertical direction, the transconjugants increased with the biofilm growth, proving VGT played an important role in the following transfer processes after HGT happened. These results were consistent with 2D observation of HGT and VGT in our previous studies [34,35]. Moreover, other studies detected low overlap between the enriched genes and HGT datasets and suggested that most functions that bacteria rely on to survive in an *E. radiata* biofilm are driven by vertical transmission process and not horizontal evolution [36]. As VGTs were deeply influenced by the substrate supply, it should be considered as the most important factor for ARG dissemination.

**Figure 4:**
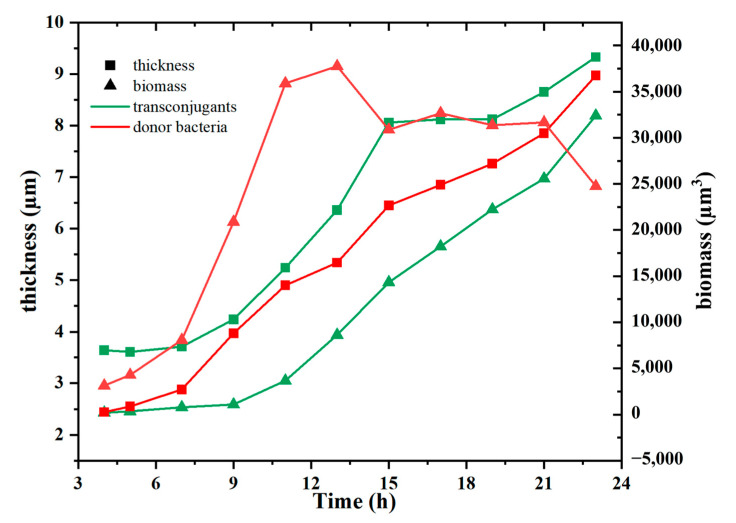
The dynamic thickness and biomass of transconjugants and donor bacteria with time.

According to Figure 4, both the biomass and thickness of transconjugants simultaneously kept increasing over time. While for the donor bacteria, the value of donor bacteria started to decrease at 9 h, at which time the transconjugants occurred, and the biofilm began to thicken. The thickness of both donor and transconjugants in the recipient bacteria increased as the time extended; however, the biomass of donor bacteria decreased after 12 h. Due to the limited space between the agarose layer and cover glass in the microfluidic chip, bacteria that could grow more quickly could become the dominant bacteria in the biofilm. According to our previous study [35], the specific growth rate of the sludge bacterial community (0.61 ± 0.08 h^−1^) was higher than donor bacteria (0.44 ± 0.06 h^−1^). Moreover, bacteria in the bottom of the biofilm were dead due to the lack of substrates. Therefore, the biomass of donor bacteria did not increase but decreased with prolonged time. Based on the results of 3D observation of ARG transfer process in the biofilm, bacterial growth rate is important for ARG spread related to VGT, implying bacteria with a high specific growth rate have great potential for ARG spread, especially when the substrate supply is high enough in the environment biofilms. As a whole, VGT involves the offspring response to replication of parental genetic materials, while HGT mainly involves the MGEs communication amongst cells, both of which are beneficial to the development of ARGs.

### 2.4. The Effect of Different Sewage Concentrations on the Transfer Frequency in Biofilms

Abundant substrate conditions exist in environments such as wastewater treatment plants. It is generally reported that the biofilm process, one of the most common wastewater treatment techniques, has been widely used in biological wastewater treatment in recent decades. Therefore, it is important to understand the effect of substrate on ARG spread in the biofilms under different sewage concentrations.

**Figure 5:**
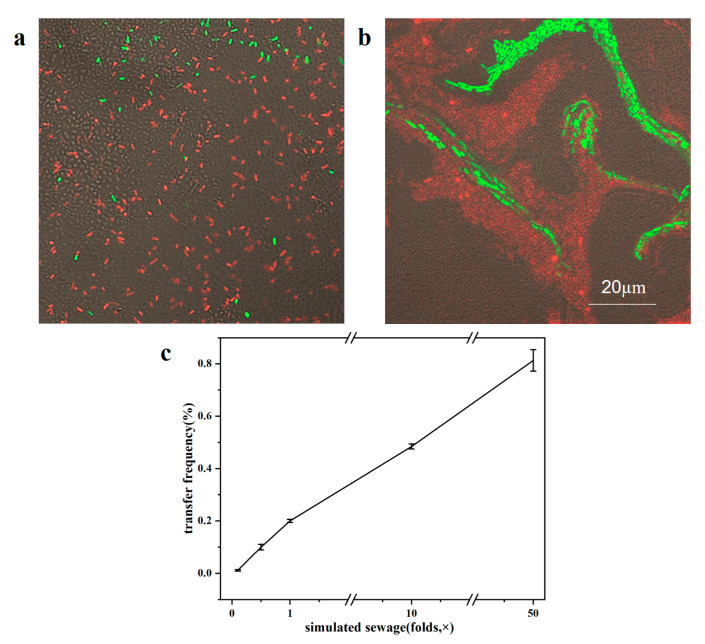
ARG transfer features under different substrate concentrations of simulated sewage. (**a**) CLSM image of biofilm with 1× simulated sewage (24 h, 100× objective, single layer); (**b**) CLSM image of biofilm with 50× simulated sewage (24 h, 100× objective, ~5 µm thickness). The scale bar represents 20 µm. The CLSM images are stacks of brightfield and fluorescent pictures; (**c**) transfer frequencies under different substrate concentrations.

In order to simulate the gradient of the substrate directly, different concentrations of simulated sewage (signed as 0.1×, 0.5×, 1×, 10×, 50×) were used to culture the mixed bacteria (donor and recipient bacteria are described as above) at 30 °C for 24 h. The CLSM images of biofilms with 1× and 50× simulated sewage on the chip are shown in Figure 5a, and the thickness of the biofilm was single layer and ~5 µm, respectively. Compared to the biofilm formed as supplied by LB medium at 24 h, the thickness was much smaller than the biofilm under the central channel (~25 µm, Figure 1), due to the relatively lower concentration of growth substrates in the simulated wastewater. The transconjugants in the biofilm with 1× simulated sewage were distributed throughout the image, while in the biofilm with 50× simulated sewage, the transconjugants flocked together, which was obviously more than that in the 0.1× biofilm.

At the same time, the well plates were used to evaluate the transfer frequencies under different sewage concentrations. The results showed that the transfer frequencies obviously increased with the increase in substrate concentration and reached the highest in the condition of 50× simulated sewage, which was 8.2 folds higher compared with the 0.5× simulated sewage (Figure 5b). Increasing the substrate concentration can improve the physiological and metabolic response of bacteria, promote the division and growth of transconjugants, and thus increase the transfer frequencies.

It has been reported that substrates are the key factors in the antibiotic resistance transfer frequency of microorganisms. Many studies performed in soil and aquatic environments have shown plasmid transfer tended to enhance in substrate-rich environments, probably because of the increased bacterial activity and cell-to-cell communication [37]. Table 1 shows that the transfer rates are higher in biofilm communities than those in planktonic states. Indeed, compared with mating in liquid culture, there are more transconjugants found in a filter. The bacteria prefer to form highly structured aggregation communities to better cope with the environmental change [38], and bacteria in biofilms show specific characteristics other than the planktonic culturation, including heterogeneity of gene expression, role assignment of the community number and the enhanced tolerance of antibiotics [39]. The typical explanation for the above phenomenon is that biofilms can promote the stability of plasmid and enhance the host range of MGEs due to the spatial and structural advantages [40].

For the ARG transfer frequencies in biofilms, they were distinct and affected by species of donor/recipient strains, experimental methods and substrate matrixes. In present studies, *E. coli* is often used as donors in conjugation experiments, and sludge and soil bacteria are used as recipients to study the realistic transfer effect. The addition of glucose and α-lactose resulted in increased transfer frequencies by over 4 and 2 orders of magnitude in the bacterial community, while sodium acetate had very little influence [46]. The comparison of transfer frequencies between rich substrates (LB medium) and common simulated natural conditions media (synthetic wastewater (SWW), soil extract (SE)) showed that about 2 and 4 logs decrease in conjugation events occurred in SWW and SE, respectively [48]. When the substrate concentration moved from 1/10 to 1/3 strength MHB, the number of transconjugants increased by ten fold; interestingly, at the same substrate condition, the bacteria with the same generation numbers have the similar transconjugants numbers [49]. In this study, the transfer frequency also increased with the increase in substrate concentration (simulated sewage); when the substrate concentration went from 0.1× to 50×, the transfer frequency increased by about 70 fold. These results also indicated the importance of pollutant control (COD, N, P, etc.) in the WWTP effluent, which normally are substrates supporting bacterial growth and discharged to the rivers or used for soil irrigation. Lower substrate conditions benefit decreasing ARG transfer frequencies in the environment. Therefore, reducing the pollutant emission is helpful for ARG control in the environment.

## 3. Materials and Methods

### 3.1. Bacterial Growth Substrates 

In this study, bacterial growth substrates mainly included a carbon source, nutrients (N, P) and inorganic salts, which are the essential compounds for bacterial growth and supplied by LB medium or simulated wastewater. The LB medium is composed of 10 g/L tryptone, 5 g/L yeast extract and 10 g/L NaCl, which are the sources of nutrients, carbon and inorganic salt, respectively. For the simulated wastewater, different concentrations were signed as 0.1×, 0.5×, 1×, 10×, 50×. Among them, 50× simulated sewage was composed by 8 g/L peptone, 0.1 g/L MgSO_4_·7H_2_O, 1.5 g/L urea, 0.35 g/L NaCl, 5.5 g/L beef extract, 0.2 g/L CaCl_2_·2H_2_O, and 1.4 g/L K_2_HPO_4_, which was used after sterilization [50].

### 3.2. Bacterial Strains and Culture

The donor strain was generously provided by Professor Barth’s group at the Technical University of Denmark. The donor strain was a genetically engineered bacteria, which was called *E. coli* MG1655 hosting a green fluorescent protein (GFP) plasmid pKJK5 with trimethoprim resistance, and this donor strain was chromosomally tagged with a gene cassette encoding constitutive red fluorescence and constitutive lacIq production. As a result, there is no GFP expression in the donor strains, but upon plasmid transfer to a new bacterium, GFP expression is possible, resulting in green fluorescent cells, which can be observed by microscope. *E. coli* MG1655 was incubated in an LB medium overnight culture with 50 mg/L trimethoprim at 30 °C.

The recipient was a complex bacterial community extracted from activated sludge, which was collected from returned sludge in a certain wastewater treatment plant (WWTP, Beijing, China). The samples were first mashed in a high-speed tissue mill (Bilon JJ-2, Shanghai Bilon Company, Shanghai, China) by intermittent crushing for 5 min every 10 min within one hour, then the mixed liquor was cultured in LB medium overnight at 30 °C for use.

### 3.3. Biofilm Formation and Mating Assays on Microfluidic Chips

The microfluidic device was fabricated as previously described [51]. The single channel was placed on the top and in the center of the agarose membrane (20 mm long and 20 mm wide) as Figure 1a shows. Donor and recipient cells were firstly washed by PBS three times and then diluted to ~10^8^ cells/mL, and the proportion of donor and recipient cells was 1:1 to obtain the initial bacterial solution used for the mating assays. A 5 μL drop of mixed bacterial solution cells were inoculated between the agarose membrane and glass. During the experiments, LB medium was continuously delivered at a speed of 2 μL/min by a syringe pump (NE-4000, NEWERA Pump Systems Inc., Farmingdale, NY, USA) into the channel of the chip for 24 h. The distribution of bacterial growth substrates in the medium was heterogeneous during the experiments, and different thicknesses of biofilms can form between the agarose membrane and glass. At the same time, ARG transfer happened during the biofilm formation, which could be recorded by the microscope. Independently repeated mating assays were performed on the chips three times.

### 3.4. Mating Assays in the Well Plate

Donor strains (*E. coli* MG1655) and recipient strains (sludge bacteria) were mixed and cultured at 30 °C for 24 h using the fully automatic growth analyzer (Bioscreen C, Helsinki, Finland). In the culture plate, there are 5 μL mixed bacteria and 195 μL simulated sewage. After the experiment, the bacterial liquids were sucked out from the plate, then washed by PBS three times to analyze transfer frequency by flow cytometry. The flow cytometry used in this study was BD LSRFortessa (BD Inc., Sussex, NJ, USA); the excitation light wavelengths were set to 488 nm and 561 nm to detect the red and green fluorescence signals. The donor strains expressed positive red fluorescence and negative green fluorescence, the recipient strains expressed both negative red and green fluorescence, but when the transfer occurred, the transconjugants expressed positive green fluorescence and negative red fluorescence. The transfer frequency was calculated by number of transconjugants/number of recipients, abbreviated to T/R [52]. Independently repeated mating assays were performed in the well plates three times.

### 3.5. Image Acquisition and Analysis

Time-lapse images were recorded by a charge-coupled device (CCD, iXon X3 897, Andor Company, Belfast, UK) through an inverted fluorescence microscope (Ti E, Nikon Corp., Tokyo, Japan) equipped with a 20×/0.45 objective lens. The observation region was between the agarose membrane and glass slip for cell tracking. Both brightfield and fluorescence images were taken during the mating assay. All images were processed with ImageJ software and auto contrast was applied to images to give clearly defined cell edges. The area of each image was 0.168 mm^2^; the proportion of transconjugants with green fluorescence (*A*_PG_) in each image at different time was calculated as Equation (1).

(1) $$A_{\mathrm{PG}}=\frac{A_{G}}{0.168}\times 100\%$$

A confocal laser scanning microscope (CLSM, LSM710, Zeiss Company, Oberkochen, Germany), equipped with 100×/1.40 oil DIC (differential interference contrast) objective lenses, was applied to the layer scan to obtain the biofilm thickness and detailed pictures of transconjugants at the end of mating assays (24 h).

Furthermore, in order to obtain the detailed information of ARG transfer during biofilm formation, a live cell imaging system (Delta Vision Elite, General Electric Company, Boston, MA, USA) was used to record the transfer process by obtaining 3D images. Imaris (v9.0, Switzerland) software was used to build the 3D model; then, through the function “biofilm analysis” of the software, the bacterial thickness (µm) and biomass (µm^3^) at different positions were obtained.

## 4. Conclusions

In conclusion, the processes of biofilm formation and ARG transfer were both tracked by 2D and 3D observation through microfluidic chips. The thickness of biofilms formed were positively related to the bacterial growth substrates due to the diffusion dynamics of the medium. The ARG transfer was triggered almost at the same time in different thicknesses of biofilms, but the following dynamic processes were totally dependent on the substrate supply in order for VGT to play the important role. Through this study, we tied the substrates, biofilm structure and transfer of antibiotic resistance together and simulated the realistic substrate conditions to investigate their influence. As biofilms attract more and more attention to bacterial evolution, the occurrence of antibiotic resistance genes and virulence genes, this study can supply more awareness about the genes transfer in different nutrient conditions of biofilms. Importantly, reducing pollutant emissions is helpful for ARG control in the environment.

## Figures and Tables

**Table 1:** Transfer frequency of ARG under different experimental methods.

Donor/Recipient	Substrates	Culture Method	Transfer Frequency (Transconjugants/Recipients)	Ref.
*E. coli* K12 (ATCC 47076)/activated sludge bacteria	COD_Cr_ 194.3–315.9 mg/L	MBR reactor	2.76 × 10^−5^	[41]
*Pseudomonas putida* KT2442/soil bacteria	Soil extract and R2A medium	Filter mating	8.24 × 10^−5^–4.56 × 10^−4^	[42]
*E. coli* MG1655/influent, effluent	LB medium	Membrane filter	5.1 × 10^−2^–7 × 10^−1^	[43]
*P. damselae*/*E. coli* K12	LB medium	Filter mating	(6.62 ± 1.61) × 10^−3^	[44]
*E. coli* HB101/*E. coli* NK5449	LB medium	Mixed cultivation	10^−5^–10^−3^	[45]
*E. coli* K12/*E. coli* NK5449	Sterile wastewater (NaAc, α-lactose, glucose)	Mixed cultivation	10^−7^–10^−4^	[46]
*E. coli* MG1655, *P. putida* KT2440/activated sludge bacteria	Synthetic wastewater medium	Filter mating	3.39 × 10^−5^–5.05 × 10^−4^	[47]
*E. coli* MG1655/*E. coli*	Synthetic wastewater, soil extract	Filter mating	3, 6 logs decrease respectively than LB medium	[48]
*E. coli* CV601/*E. coli* J53	1/10, 1/3-strength Mueller–Hinton broth(MHB)	Filter mating	10^1^–10^3^, 10^2^–10^4^ CFU/mL transconjugants	[49]
*E. coli* MG1655/sludge bacteria	Simulated wastewater with different concentrations	Mixed cultivation	10^−4^–10^−3^	This study

## Data Availability

The data presented in this study are contained within the article.
